# Design of highly nonlinear confusion component based on entangled points of quantum spin states

**DOI:** 10.1038/s41598-023-28002-7

**Published:** 2023-01-19

**Authors:** Hafiz Muhammad Waseem, Seong Oun Hwang

**Affiliations:** 1 Department of IT Convergence Engineering, Gachon University, Seongnam, South Korea; 2 Department of Computer Engineering, Gachon University, Seongnam, South Korea

**Keywords:** Applied mathematics, Computational science, Computer science, Information technology

## Abstract

Cryptosystems are commonly deployed to secure data transmission over an insecure line of communication. To provide confusion in the data over insecure networks, substitution boxes are the solitary components for delivering a nonlinear mapping between inputs and outputs. A confusion component of a block cipher with high nonlinearity and low differential and linear approximation probabilities is considered secure against cryptanalysis. This study aims to design a highly nonlinear substitution-permutation network using the blotch symmetry of quantum spin states on the Galois field *GF* (2^8^). To observe the efficiency of the proposed methodology, some common and advanced measures were evaluated for performance, randomness, and cryptanalytics. The outcomes of these analyses validate that the generated nonlinear confusion components are effective for block ciphers and attain better cryptographic strength with a high signal-to-noise ratio in comparison to state-of-the-art techniques.

## Introduction

The rapid growth of multimedia communication necessitated the requirement for safe and contemporaneous transmission and information exchange. A prominent approach to cope with this is to consider a plain bit stream and apply either modern or traditional cryptographic standards, such as the Data Encryption Standard (DES)^1,2^, Advanced Encryption Standard (AES)^3,4^, and International Data Encryption Algorithm (IDEA)^5,6^. Modern block ciphers (DES and AES) depend on Shannon’s theory of confusion and diffusion^7^. Confusion refers to the practice of generating the relationship between key and ciphertext as complex as possible, whereas, the influence of a single bit on multiple cipher bits to opaque the statistical redundancies of plaintext refers to diffusion.

The substitution box (S-box) is a vectorial Boolean function that maps $F_{2}^{n}$ to $F_{2}^{m}$, where $F_{2}^{n}$ signifies Galois field $GF\left( {2^{n} } \right)$^8^. It is the solitary component in a block cipher to deliver confusion through nonlinear mapping between the inputs and outputs to perceive the practice of encryption. There have been numerous solicitations of S-boxes available in the literature for image encryption^9–11^, low-profile mobile applications^12^, multimedia encryption^13^, watermarking, and steganography^14^.

Linear and differential cryptanalyses are based on the probabilistic characteristics of the cipher parameters and output. These are considered powerful attacks on block ciphers. These attacks signify the strength of the encryption algorithm by increasing the number of rounds in the structure^15^. The nonlinearity of S-boxes causes uncertainty in the output, which provides resistance against differential and linear attacks^16,17^. An S-box with high nonlinearity and low linear and differential probabilities is always favorable for a cryptosystem^18^. Therefore, the design of such components with good cryptographic properties plays a significant role in cryptographic applications^19^.

Substitution permutation network (SPN) structures are commonly implemented in AES and Feistel types of networks, such as DES^20^. AES uses bijective components to assemble the system invertible, whereas the DES approach is not limited to bijectivity. For instance, the proposed PICARO method^21^ uses non-bijective mapping in Feistel networks. However, the nonlinearity using the PICARO method reached 94, which is far from the Rijndael optimal value of 112 in AES.

True random classifications for cryptography; however, have been validated by researchers since techniques rely on the robustness of naturally arising mechanisms to generate true randomness^22–25^. These types of sequences are non-reproducible, unpredictable, and irreversible, all the while their internal assembly and response history are understood by adversaries. Quantum spin states, maps, and chaos exhibit the favorable properties of capriciousness, ergodicity, control parameters, and sensitivity to the initial value(s) that fulfill the requirements of confusion and diffusion properties for the cryptosystem(s)^26,27^.

### Problem statement

With the advent of quantum technology, several traditional security standards and cryptographic solicitations may be easily exploited and mistreated^28–30^. However, except for some attacks such as meet-in-middle and side-channel^31,32^, no better attacks exist other than brute-force to exploit the weakness in key scheduling and inadequate diffusion features in SQUARE^33^ and AES^4^, which entail millions of years for decryption. However, quantum classifications have attracted great attention in scientific and engineering disciplines, especially in the design of new cryptosystems and cryptanalyses, which is a threat to AES and Feistel network-based applications by performing reverse computation or executing brute force using quantum computation. As the confusion component is considered an intense constituent for most of the assemblies to resist attacks, many researchers laid their potential to improve existing as well as proposing new structures either with the traditional or by using quantum practices to generate secure S-boxes^34–36^. To resist classical and quantum attacks, there is a need to design a substitution permutation structure with highly nonlinear confusion components and comparable cryptographic properties for classical standards.

### Related work

Numerous structures exist to generate the outcomes for S-boxes with either traditional or modern chaos-based algorithms^37,38^, and many researchers have applied optimization methods, such as evolutionary algorithms^39–41^, to improve the chaos-based consequences for the confusion component. Although these methodologies to design the structures of S-boxes offer favorable characteristics, researchers have also pointed out the weaknesses of these approaches^42^. Many statistical attacks are available for the assembly of S-box designs, including linear and differential^43–45^, interpolation^46^, Grobner basis^47^, side-channel^48^, SAT solver^49^, XL^50^, and XSL^51^ attacks. Chaos-based systems have been used extensively in the construction of confusion components^52,53^, but owing to the inherent algorithmic advancement of control parameters and periodicity in the maps, several weaknesses of these systems also exist in the literature, including discontinuity and non-uniform distribution in chaotic sequences^54,55^, predictability^56,57^, finite precision effect and short quantity of randomness^58,59^, dynamical degradation of chaotic systems and frail chaos^60,61^, and a small number of control parameters^62,63^.

The authors in^64^ generated the outcomes by transforming the binary Gray code into a standard AES S-box. Likewise, the authors in^65^ offered two boxes for AES, and the authors in^66^ minimized the computational complexity of AES by modifying the affine transformation matrices. A few recent studies for designing dynamic S-boxes using cellular automata (CA) are highlighted in^67–69^ with comparable cryptographic properties. However, these practices do not provide substantial attributes because of the insignificant differential probability values^70^.

Among the computational prototypes established in the quantum era, quantum walks^71,72^, quantum spin states^73,74^, and quantum chaos^75,76^ have been employed to develop modern algorithms. The generated confusion components using these procedures still need improvements to compete with standard algorithms, such as AES, in the sense of nonlinearity and balancedness. Although, the physical hardware for quantum computing is not yet available, the inspired frameworks provide platforms for emulating pseudo-quantum algorithms, which can perform various quantum mechanical solicitations endorsed by the influence of quantum computations within the constraints executed by the capability of classical machines^77^.

### Contribution

Inspired by the tremendous nonlinear features of quantum algorithms, the constraints of classical cryptosystems can be enhanced by designing state-of-the-art projections for effective applications in information security^78–81^. The main contribution of this research is to explore the assimilation of quantum-inspired algorithms into conventional cryptographic applications. To accentuate the highly nonlinear balanced S-boxes (8, 8) described by the property of having an exceptionally high resistance to linear and differential cryptanalysis, we developed a bit-level quantum dot protocol on the blotch symmetry of quantum spin states to generate the true random sequence. We also designed and used a white-box to map multiple bits within a single state into a singular bit to balance the output of each state. It also resists the reverse engineering process to cope with brute-force attacks performed either in classical or quantum machines.

The confrontation of linear and differential cryptanalysis for the evaluated nonlinear components is superior to some common boxes used in AES, APA^82^, Gray^64^, PICARO^21^, NSA’s Skipjack^83^, and state-of-the-art block ciphers. The maximum nonlinearity achieved using the Rijndael structure for AES is 112 and 94 in PICARO, whereas our method generates S-boxes with a nonlinearity of 114.

To estimate the efficiency of the projected model, we compared the numerical evaluation of confusion components with well-established criteria of some state-of-the-art mechanisms, such as nonlinearity, balancedness and bijectivity, linear and differential approximation probabilities (LP and DP), strict avalanche and bit independence criteria (SAC and BIC), NIST statistical suite, and cryptanalytic analyses. The results of the proposed methodology validate that the generated S-boxes are feasible for multifaceted solicitations in information security.

## Methodology

Collection of Boolean functions $F\left( {X_{n} } \right) = \left( {f_{1} \left( {X_{n} } \right),...,f_{m} \left( {X_{n} } \right)} \right)$ through mapping of $F:{\mathbb{F}}_{2}^{n} \,a\,{\mathbb{F}}_{2}^{m}$ over the Galois field $GF\left( {p^{m} } \right)$^84^ to generate confusion component using the blotch symmetry of quantum states is evaluated in this section. The details of Boolean Functions, Galois Field, and the Substitution box are provided in the supplementary information underneath the heading preliminaries. This section provides a brief overview of the mechanism used to construct the confusion components, such as the evaluation of quantum dots from states, the development of balanced Boolean function values, the pseudo algorithm, and the structural flowchart.

### Evaluation of Quantum dots

The unitary group of degree $n$ ‘$SU(n)$’ is defined as a set of $n \times n$ special matrices with entries from complex numbers having determinant one. It can be signified as:

$$SU(2) = \left( {\begin{array}{*{20}c} {z_{1} } & { - \overline{z}_{2} } \\ {z_{2} } & {\overline{z}_{1} } \\ \end{array} } \right)$$

where $$z_{1} ,z_{2} \in C$$, and $$\,\left| {z_{1} } \right|^{2} + \left| {z_{2} } \right|^{2} = 1$$.

The eigenvalue of the physical observable spin system $$S_{z}$$ involves the relationship of $$\pm \hbar /2$$, kets $$\left| {\left. \pm \right\rangle } \right.$$, and the operators in a system of spin $${\raise0.5ex\hbox{$\scriptstyle 1$} \kern-0.1em/\kern-0.15em \lower0.25ex\hbox{$\scriptstyle 2$}}$$ are $$S_{z} \left| {\left. + \right\rangle } \right. = + \frac{\hbar }{2}\left| {\left. + \right\rangle } \right.$$ and $$S_{z} \left| {\left. - \right\rangle } \right. = - \frac{\hbar }{2}\left| {\left. - \right\rangle } \right.$$.

We consider $S_{z}$ as the most general form of the $2 \times 2$ matrix as:

$$S_{z} = \left( {\begin{array}{*{20}c} p & q \\ r & s \\ \end{array} } \right)$$

Therefore, the spin $${\raise0.5ex\hbox{$\scriptstyle 1$} \kern-0.1em/\kern-0.15em \lower0.25ex\hbox{$\scriptstyle 2$}}$$ system can be engraved as: $$\left( {\begin{array}{*{20}c} p & q \\ r & s \\ \end{array} } \right)\left( {\begin{array}{*{20}c} 1 \\ 0 \\ \end{array} } \right) = + \frac{\hbar }{2}\left( {\begin{array}{*{20}c} 1 \\ 0 \\ \end{array} } \right)$$, and $$\left( {\begin{array}{*{20}c} p & q \\ r & s \\ \end{array} } \right)\left( {\begin{array}{*{20}c} 0 \\ 1 \\ \end{array} } \right) = - \frac{\hbar }{2}\left( {\begin{array}{*{20}c} 0 \\ 1 \\ \end{array} } \right)$$

The generated solutions to the above equivalences are: $p = + \frac{\hbar }{2}\,\,\,,\,\,\,q = 0,\,\,\,r = 0,\,\,{\text{and}}\,\,s = - \frac{\hbar }{2}.$

The corresponding outcomes for the above solution are: $$S_{z} = \frac{\hbar }{2}\left( {\begin{array}{*{20}c} 1 & 0 \\ 0 & { - 1} \\ \end{array} } \right)\,\,\,\,\,\left| {\left. + \right\rangle } \right. = \left( {\begin{array}{*{20}c} 1 \\ 0 \\ \end{array} } \right)\,\,\,\,\,\left| {\left. - \right\rangle } \right. = \left( {\begin{array}{*{20}c} 0 \\ 1 \\ \end{array} } \right)$$.

Therefore, the spin operators in the $x,\,\,y$ and $z$ directions with eigenvalues of $\pm \,\hbar /2$ are evaluated as.

$$S_{x} = \frac{\hbar }{2}\left( {\begin{array}{*{20}c} 0 & 1 \\ 1 & 0 \\ \end{array} } \right)\,\,\,\,\,\,\left. {\left| + \right.} \right\rangle_{x} = \frac{1}{\sqrt 2 }\left( {\begin{array}{*{20}c} 1 \\ 1 \\ \end{array} } \right)\,\,\,\,\,\,\left. {\left| - \right.} \right\rangle_{x} = \frac{1}{\sqrt 2 }\left( {\begin{array}{*{20}c} 1 \\ { - 1} \\ \end{array} } \right)$$,

$$S_{y} = \frac{\hbar }{2}\left( {\begin{array}{*{20}c} 0 & { - i} \\ i & 0 \\ \end{array} } \right)\,\,\,\,\,\,\left. {\left| + \right.} \right\rangle_{y} = \frac{1}{\sqrt 2 }\left( {\begin{array}{*{20}c} 1 \\ i \\ \end{array} } \right)\,\,\,\,\,\,\left. {\left| - \right.} \right\rangle_{y} = \frac{1}{\sqrt 2 }\left( {\begin{array}{*{20}c} 1 \\ { - i} \\ \end{array} } \right)$$, and.

$$S_{z} = \frac{\hbar }{2}\left( {\begin{array}{*{20}c} 1 & 0 \\ 0 & { - 1} \\ \end{array} } \right)\,\,\,\,\,\,\left| {\left. + \right\rangle_{z} } \right. = \frac{1}{\sqrt 2 }\left( {\begin{array}{*{20}c} 1 \\ 0 \\ \end{array} } \right)\,\,\,\,\,\,\left| {\left. - \right\rangle_{z} } \right. = \frac{1}{\sqrt 2 }\left( {\begin{array}{*{20}c} 0 \\ 1 \\ \end{array} } \right)$$.

The constituent of the spin system in a direction along the unit vector $\hat{n}$ is ${\hat{\mathbf{n}}} = {\hat{\mathbf{i}}}\sin \theta \cos \phi + {\hat{\mathbf{j}}}\sin \theta \sin \phi + {\hat{\mathbf{k}}}\cos \theta$.

Therefore, the spin vector ${\mathbf{S}}$ can be transformed into a new unit vector $S_{n} = {\mathbf{S}} \cdot {\hat{\mathbf{n}}}$, hence,

$S_{n} = S_{x} \sin \theta \cos \phi + S_{y} \sin \theta \sin \phi + S_{z} \cos \theta$, or more generally, $$S_{n} = \frac{\hbar }{2}\left( {\begin{array}{*{20}c} {\cos \theta } & {\sin \theta e^{ - i\phi } } \\ {\sin \theta e^{ - i\phi } } & { - \cos \theta } \\ \end{array} } \right)$$, and the eigenvectors are $\left. {\left| + \right.} \right\rangle_{n} = \cos \frac{\theta }{2}\left. {\left| + \right.} \right\rangle + \sin \frac{\theta }{2}e^{i\phi } \left. {\left| - \right.} \right\rangle$ and $\left. {\left| - \right.} \right\rangle_{n} = \sin \frac{\theta }{2}\left. {\left| + \right.} \right\rangle - \cos \frac{\theta }{2}e^{i\phi } \left. {\left| - \right.} \right\rangle$.

**Figure 1:**
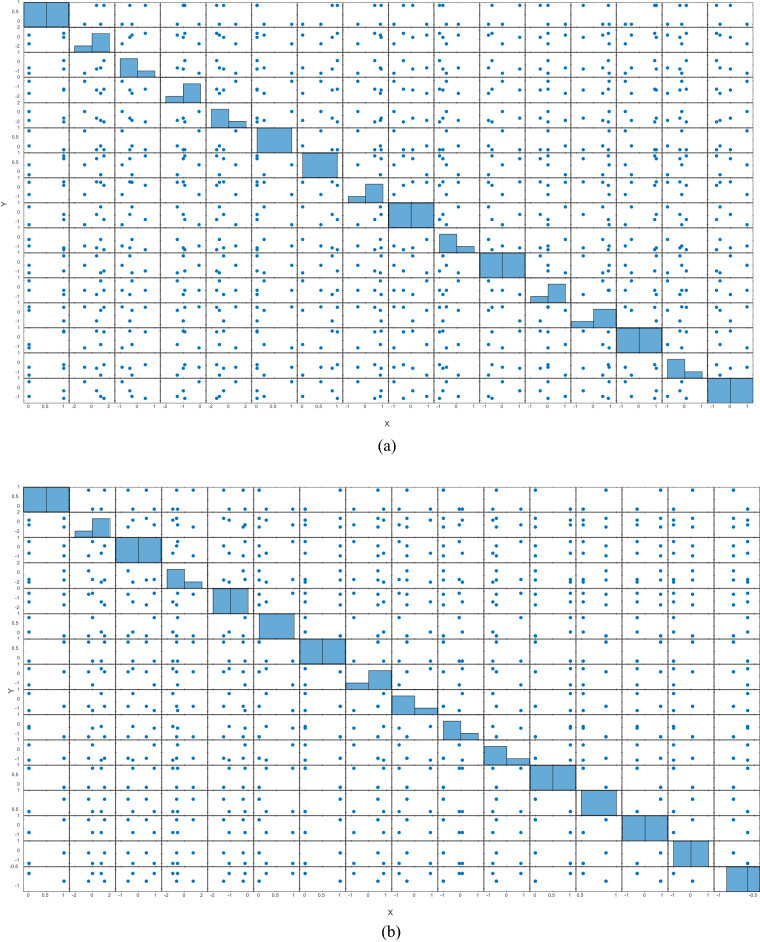

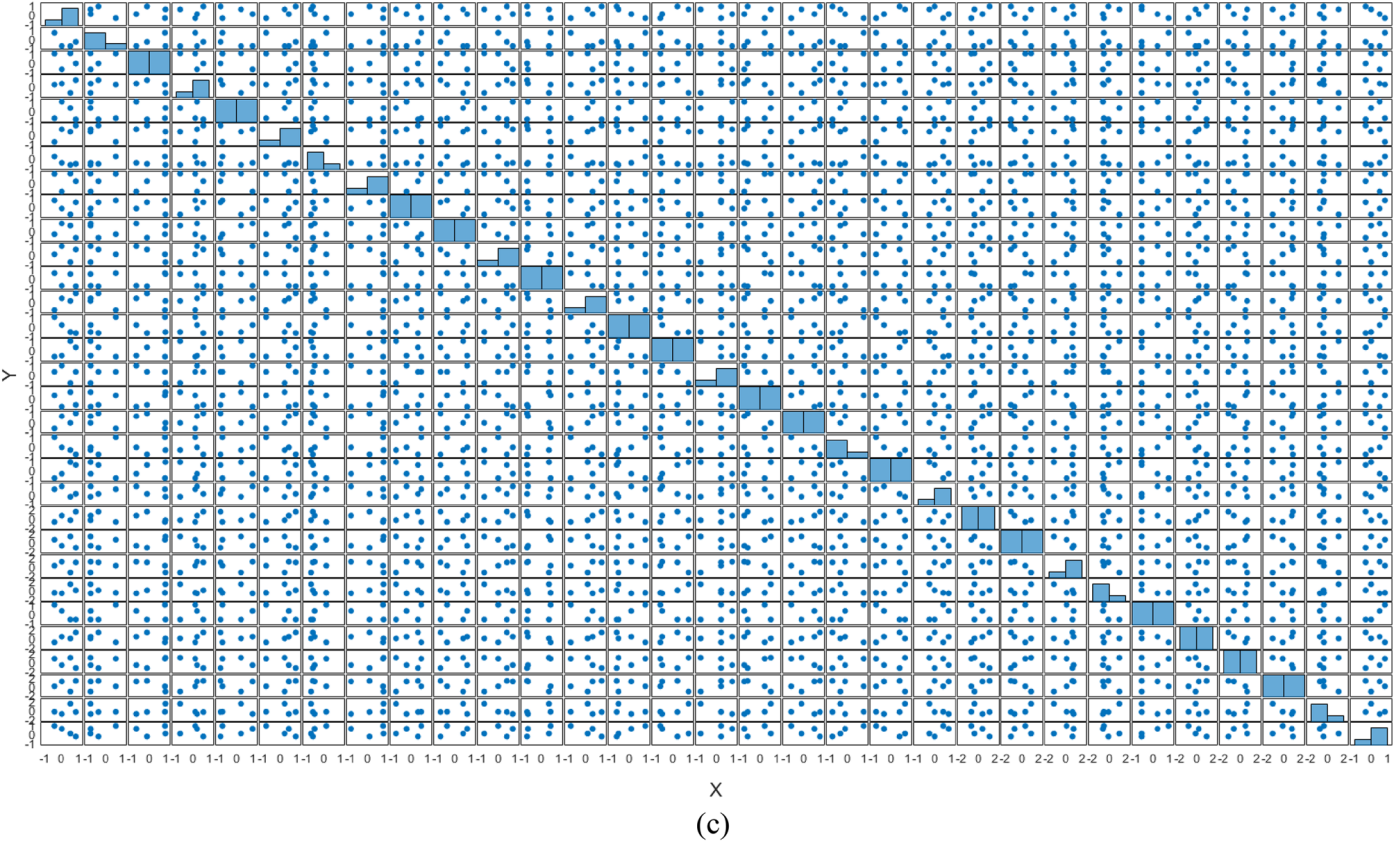
Evaluation of quantum dots at the entangled point of spin states. (**a**) Extraction of dot points at the 8th entangled state in the phase domain of 0 to 360 with a step size of 90, (**b**) Extraction of dot points at the 14th entangled state in the phase domain of 0 to 360 with a step size of 90, (**c**) Extraction of dot points at the 1st entangled state in the phase domain of − 90 to 90 with a step size of 20.

Let us entangle the produced $2 \times 2$ matrices from $S_{n}$ in the $x,\,\,y$ and $z$ directions by introducing the identity matrix to generate a set $S$ of $4 \times 4$ entangled matrices, that is, $S = \{ S_{k} \in S_{4 \times 4} \,(I,S_{x} ,S_{y} ,S_{z} \,),\,\,k = 1,2,...,24\,.\}$. The points at which the entangled states reflect their symmetry are referred to as quantum dots^85^, as shown in Fig. 1.

### Generation of balanced Boolean function values

**Figure 2:**
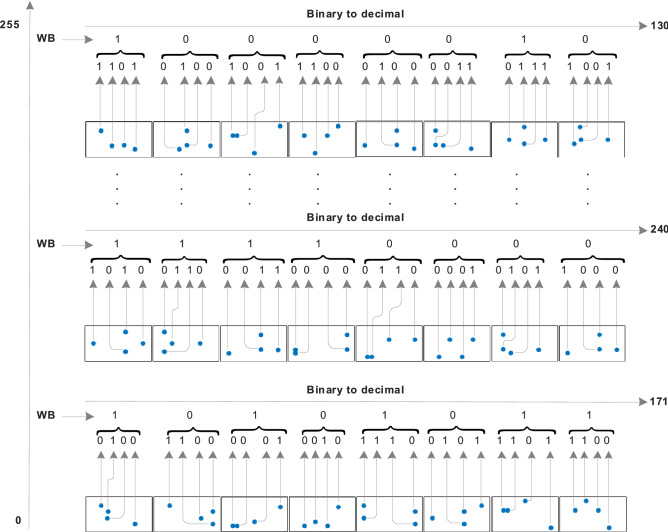
Demonstration of Quantum dots in entangled states to produce binary numbers and their decimal values for the confusion component.

**Figure 3:**
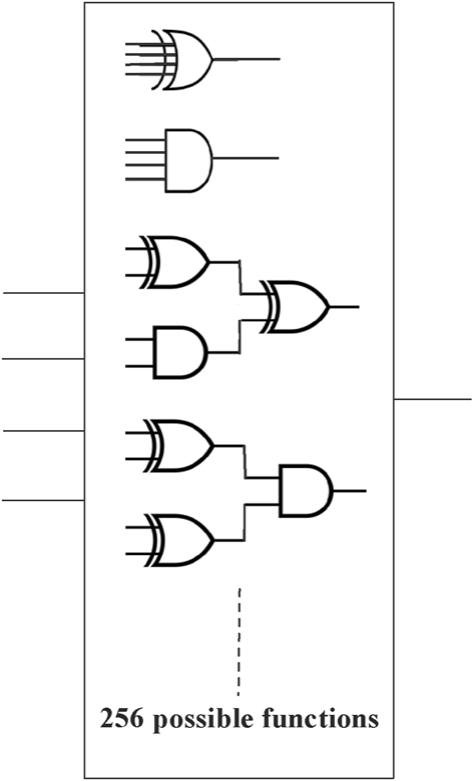
Possible Boolean functions for the mapping of 4 bits to a 1-bit value.

The generation of four bits in each state in Fig. 2 is mapped to a single bit using white-box (WB) with multiple operations that map ${\mathbb{F}}_{2}^{4}$ to ${\mathbb{F}}_{2}$, as demonstrated in Fig. 3, to evaluate the binary sequence(s) to generate S-boxes. The total number of functions in the white-box that correspond to input bits will be $2^{{2^{n} }}$, implying that there will be 256 Boolean functions in the white-box for four input values.

If the produced 8-bit sequence is similar to the previous state(s) sequence or unable to satisfy the balance criterion, the produced sequence from the same states will again operate with the WB until the condition is fulfilled. The random assortment of each operation in the WB to produce a single bit also resists the reverse engineering threats to cryptographic structures.

### Algorithm

The detailed algorithm to design highly nonlinear confusion component(s) is explained as follows:

- To initialize the setup, we first set the phase domain between − 720° and 720° with a sufficient step size $i$. A larger phase domain with a smaller step size leads to unbounded or measureless states generation.
- Each state contains multiple points to produce binary data, where the points in each state(s) will be at different positions and have distinct classifications among other states to produce truly random data.
- The algorithm of Table 1 takes statistics from the first eight states and produces an 8-bit sequence using the WB. There will be a mapping of multiple points within a state to generate a single bit using WB.
- For 256 operations with multiple of eight states, the statistics are fetched from 2048 states. If any of the 8-bit outputs using WB are similar to any previous record, the operation will be repeated in the same states until a unique sequence is generated.
- The algorithm then substantiates the balancedness property of the unique sequence. It repeats the same constraints if the condition is not fulfilled.
- It generates the S-box after validating the desired nonlinearity, SAC, and linear and differential probability approximations. It will take a designated phase shift in the input domain and perform all the operations again if any of the trials are not satisfied.

**Table 1:**
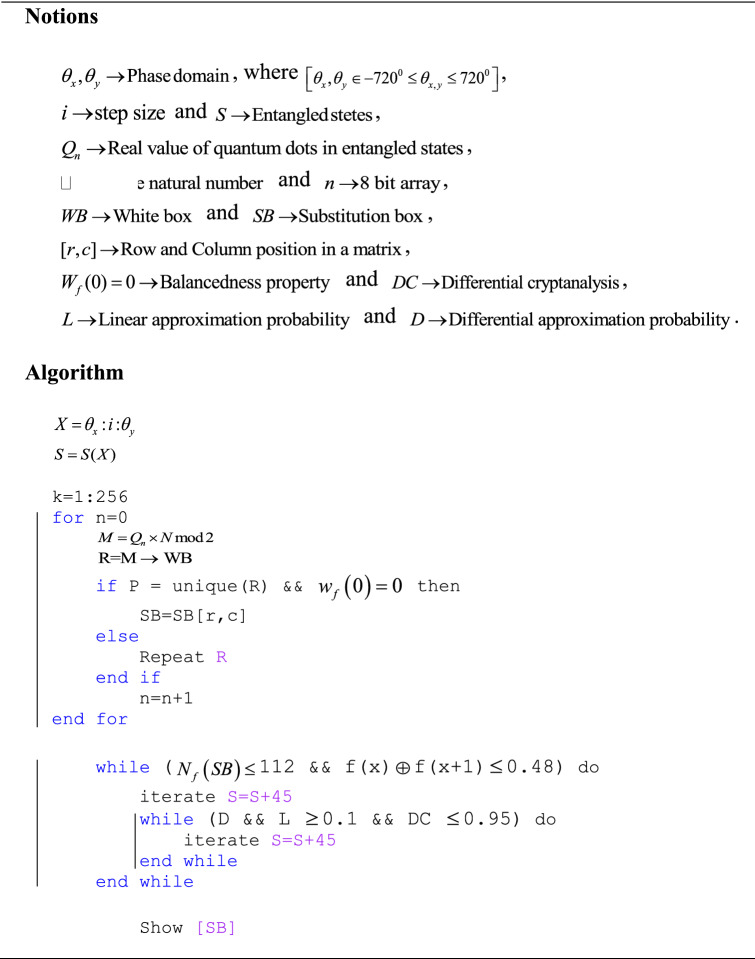
Algorithm for designing of S-box.

### Structural flowchart

**Figure 4:**
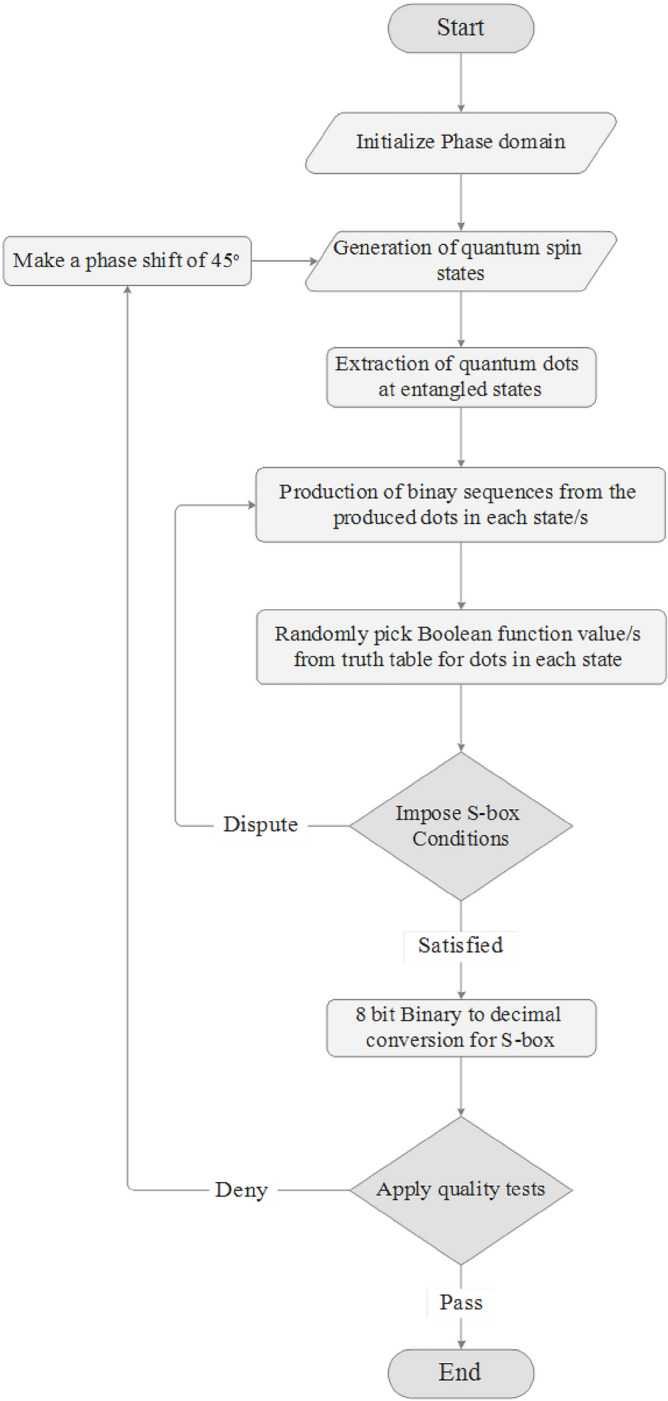
Flowchart to extract the highly nonlinear confusion component(s).

The protuberant structure used to construct the highly nonlinear balanced S-boxes for block ciphers is shown in Fig. 4. There is no prerequisite of Add-Round-Key to alter or modify the keys or phase information for different rounds in a block cipher. If there is a small phase domain to design the states for quantum dots, then after producing sufficient outcomes for the S-boxes, the states are shifted to 45° and dots are generated at different positions in each state and perform the desired operations.

S-box conditions: If there is a dispute between bits’ repetition(s) or the balancedness is not satisfied, the algorithm repeats itself on the same constraints. It will not move to the next state unless the conditions are fulfilled. The S-box is assembled after scrutinizing the conditions of the desired nonlinearity and strict avalanche quality criteria.

## Experimental results

The generated substitution boxes are listed in Tables 2 and 3 after satisfying certain criteria, such as the 0/1 balance test, bits alteration to reach the nearby affine function, sensitivity of avalanche deviations by a single variation in the network's input, minute alteration in the input sequence to observe the variation in the output, and determination of the maximum disparity of the event's outcome by witnessing the imbalance between the input and output bits^86^. The supplementary information contains a comprehensive description of these analyses underneath the evaluation criteria for s-boxes. The outcomes based on the proposed methodology, as well as their assessments with standard structures and state-of-the-art implications, are discussed in the following subsections.

**Table 2:** Evaluated S-box ‘S_1_’ with the aforementioned methodology.

S_2_	0	1	2	3	4	5	6	7	8	9	A	B	C	D	E	F
0	163	123	187	201	10	85	209	193	169	26	36	38	191	114	246	13
1	74	115	226	241	58	211	53	178	100	147	245	215	255	220	203	78
2	222	122	162	152	217	135	179	165	40	186	160	69	86	103	97	12
3	167	51	83	45	231	88	126	182	55	113	30	16	208	224	183	73
4	138	145	110	176	249	91	251	19	150	219	173	161	140	57	34	238
5	159	79	80	218	7	117	101	189	137	24	205	23	65	190	47	64
6	202	5	174	50	148	247	168	177	170	248	233	228	130	84	1	105
7	39	129	92	132	127	180	41	210	252	14	164	54	144	46	21	0
8	76	81	37	17	22	243	61	141	156	108	149	239	118	204	139	221
9	43	244	206	67	98	212	116	254	146	194	124	128	42	33	104	188
A	192	35	175	227	120	111	225	235	3	56	9	66	214	237	236	2
B	29	250	94	185	200	199	70	20	230	153	157	4	196	216	63	155
C	229	77	49	99	121	60	213	240	71	87	75	198	242	44	112	181
D	207	158	11	62	31	90	166	106	107	18	27	119	95	72	172	59
E	68	52	93	234	32	133	143	48	154	171	28	195	89	142	253	8
F	184	232	6	109	102	125	223	96	82	131	136	25	15	151	197	134

**Table 3:** Evaluated S-box ‘S_2_’ with the aforementioned methodology.

S_1_	0	1	2	3	4	5	6	7	8	9	A	B	C	D	E	F
0	171	240	96	59	250	226	111	142	60	54	48	219	41	115	218	75
1	135	249	94	63	191	164	199	12	169	192	243	203	104	255	237	166
2	144	87	242	119	239	194	120	183	148	50	230	77	83	109	38	152
3	193	44	42	154	18	43	234	232	23	124	92	110	80	217	246	136
4	131	105	90	103	175	185	26	184	138	212	180	160	204	225	36	198
5	190	31	161	81	159	100	78	79	47	133	107	118	134	53	69	238
6	129	132	57	19	98	195	158	220	208	102	95	68	222	28	189	52
7	29	30	45	108	162	70	151	197	55	97	163	86	254	247	213	56
8	206	5	9	24	170	252	145	40	155	16	233	11	113	221	231	35
9	127	114	89	106	67	253	209	228	248	13	85	116	200	245	39	101
A	147	196	25	73	0	32	61	46	168	211	1	125	37	14	244	51
B	2	4	10	20	22	174	49	17	207	121	143	216	139	215	202	179
C	187	62	15	146	223	64	76	205	172	34	91	165	188	241	117	137
D	141	177	227	210	122	27	112	88	3	71	128	167	6	186	201	84
E	173	65	153	72	126	58	149	224	236	7	99	123	178	33	176	74
F	150	214	21	251	157	93	140	66	8	182	235	156	181	229	82	130

### Generated S-boxes

We evaluated multiple balanced Boolean functions, and the outcomes of the two nonlinear components for block ciphers are demonstrated in Tables 2 and 3.

The S-boxes represent a distinct group of $n$ variable Boolean functions. Therefore, we carried out the following measures to assess the performance of the intended nonlinear components. We validated the randomness in the bit stream generated with blotch symmetry of entangled states over the 2-D plane using the NIST statistical test suite (800-22)^87^. This suite entails multiple assessments, provided in the supplementary information, to investigate the security of the anticipated design. We conducted cryptanalytic analysis, including algebraic degree, absolute indicator, algebraic immunity, correlation immunity, propagation criteria to ensure the diffusion characteristics in Boolean functions, differential uniformity, differential cryptanalysis, differential power analysis in terms of signal-to-noise ratio, and transparency order to quantify the resistance of the s-boxes against differential attacks.

### Nonlinearity comparison

Linear mapping of input vectors to the output renders the cryptosystem breakable. A higher nonlinearity of the confusion component is always desirable to make the cryptosystem strong enough to provide resistance against linear and differential attacks. The nonlinearity of all Boolean function constituents for the evaluated S-boxes ***S***_***1***_ and ***S***_***2***_ and their comparison with various methodologies are given in Table 4.

**Table 4:** Evaluation of nonlinearities for generated S-boxes and comparison with benchmark approaches.

S-box	*f*_*0*_	*f*_*1*_	*f*_*2*_	*f*_*3*_	*f*_*4*_	*f*_*5*_	*f*_*6*_	*f*_*7*_
***S***_***1***_	114	114	114	114	114	114	114	114
***S***_***2***_	114	114	114	114	114	114	114	114
Zhang^8^	108	110	108	110	108	108	110	108
El-Latif^26^	104	106	108	106	106	102	108	104
Wang^34^	110	110	112	110	110	110	110	110
Ibrahim^68^	108	106	110	108	108	108	106	108
Alghafis^74^	112	111	112	111	112	112	112	112
W. Gao^84^	106	108	106	108	106	106	106	106
Jakimoski^88^	104	100	106	102	104	102	104	104
AES	112	112	112	112	112	112	112	112
APA	112	112	112	112	112	112	112	112
Gray	112	112	112	112	112	112	112	112

The maximum non-linearity for 8-bit balanced Boolean functions achievable with standard AES, APA, or Gray S-box is 112. The confusion components, either with this nonlinearity or nearby, are generally considered to be secure. We reached nonlinearity of 114 for 8-bit balanced Boolean functions with the proposed methodology, which validates superior capacity to state-of-the-art findings in resisting attacks.

### SAC and BIC comparison

A cryptographic hash function or block cipher must launch the avalanche effect to a substantial degree for reliable randomization to protect the algorithm from the cryptanalyst breaking partially or entirely by predicting the input at a given output. It is satisfied when each of the output bits flips with a 50% probability by complimenting a single input bit. The pairwise independence between avalanche variables for a specified set of avalanche vectors is evaluated here with the BIC assessment given in Table 5.

**Table 5:** Evaluation of SAC and BIC for generated S-boxes and comparison with benchmark approaches.

S-box	SAC			BIC-NL			BIC-SAC		
	Min	Max	Mean	Min	Max	Mean	Min	Max	Mean
***S***_***1***_	0.4638	0.5521	0.5000	114	114	114	0.4733	0.5217	0.5031
***S***_***2***_	0.4493	0.5419	0.5000	114	114	114	0.4816	0.5356	0.5039
Zhang^8^	0.39	0.56	0.49	–	–	94	–	–	–
El-Latif^26^	–	–	0.4958	–	–	103.93	–	–	0.5023
Wang^34^	0.4219	0.5781	0.4953	–	–	104.07	–	–	0.5021
F. Khan^42^	0.3906	0.5937	0.5031	–	–	110	–	–	0.499
Ibrahim^68^	0.4375	0.5781	0.0781	–	–	–	0.4863	0.5273	0.0273
Siddiqui^70^	0.4375	0.5625	0.5053	112	112	112	0.4863	–	0.5013
Alghafis^74^	0.4375	0.5703	0.5005	–	–	111.64	0.4844	0.5089	0.4994
W. Gao^84^	0.4063	0.5781	0.4990	98	108	103.57	0.4668	0.5	0.5033
Jakimoski^88^	0.42	0.59	0.49	–	–	–	–	–	–
AES	0.4531	0.5625	0.5049	112	112	112	0.4805	0.5280	0.5046
APA	0.437	0.562	0.499	112	112	112	0.472	0.526	0.499
Gray	0.437	0.562	0.499	110	112	111.46	0.478	0.526	0.502

By analyzing the outcomes reported in Table 5, the proposed approach produces better outcomes than state-of-the-art attainments. The maximum BIC-NL value obtained in existing methods is 112, however we reached 114 in our trial. The findings of the aforementioned method also satisfy the SAC analysis, yielding a near-optimal value of 0.5.

### NIST statistical analyses

To investigate the security of the proposed design, we executed the NIST statistical test suite (800-22) on the generated random sequence by quantum dots for S-boxes. The outcomes of this trial are presented in Table 6.

**Table 6:** Evaluation of NIST statistical test suite on the generated S-boxes and comparison with benchmark approaches.

Statistical test	*p-*values				
	*S*_*1*_	*S*_*2*_	El-Latif^26^	Wang^35^	Mahmood Malik^69^
Frequency (Mono-bits)	0.5054	0.5517	0.2918	0.699313	0.5082
Block frequency	0.5988	0.4903	0.6936	0.834308	0.8811
Run	0.6164	0.5692	0.3849	0.289667	0.6444
Longest run	0.3168	0.4401	0.1371	0.249284	0.0142
Rank	0.0311	0.0712	0.0587	0.071177	0.6738
Spectral (FFT)	0.2985	0.3630	0.3040	0.096578	0.3652
Periodic	0.5106	0.5583	0.2151	0.883171	0.5696
A-periodic	0.1869	0.2274	0.0790	0.971699	0.2453
Universal statistical test	0.0827	0.07611	0.6126	0.455937	0.0340
Linear complexity	0.1003	0.1214	0.1071	0.574903	0.8618
Serial	0.6513	0.7291	0.9145	0.964295	0.7623
Approximate entropy	0.9882	0.9615	0.0120	0.474986	0.9350
Cumulative sum	0.5186	0.4857	0.0656	0.534146	0.5770
Random excursion	0.4619	0.5483	0.1256	0.699313	0.5793
Random excursion variant	0.6120	0.5234	0.5066	0.455937	0.4476

Based on the findings in Table 6, we discovered that the produced sequence for mono-bits and block frequency assessments meet the intimacy of the ones fraction to about one-half, which exhibited that the number of ones and zeros in a sequence and in *m*-bit block(s) are approximately the same. To observe the fluctuations between the substrings, run and longest run assessments were performed, whilst the rank of disjoint sub-matrices for the entire sequence is assessed to determine the linear dependency. We evaluated the FFT to detect periodic features in the sequence to identify the divergence from the notion of randomness. Runs of zeros were not analyzed individually for periodic and a-periodic assessments because of concerns about statistical impartiality. Using a feedback register, we conducted a universal statistical test and a linear-complexity test to determine the number of bits between matching patterns and the complexity of the sequence. We also observed the frequency of each overlapping pattern in the sequence using a serial test and used approximate entropy assessment to relate the frequency of overlapping blocks to the expected outcomes for the random sequence. We evaluated the cumulative sum to determine the maximum excursion of the random walk and validated the randomness in sequence based on the findings shown in Table 6.

### Balancedness, Bijectivity, LP, and DP comparison

A function with significant disparity can easily be approximated by a constant function, and an S-box is considered balanced if all of its Boolean function constituents are balanced. To analyze the imbalance between the input and output bits and determine the maximum disparity estimation of the event’s outcome, we evaluated its linear probability. The S-boxes are considered secure against linear cryptanalysis if they have a small linear probability. For the variation in output for a minute alteration in the input sequence, we computed the differential probability. The immunity of the S-box to differential cryptanalysis is better if the maximum value of DP is as small as possible. Table 7 comprises analyses of balancedness, bijectivity, number of fixed points, LP, and DP for the assessed S-boxes using the proposed methodology, as well as comparisons with AES, APA, Gray, and state-of-the-art practices.

**Table 7:** Evaluation of balancedness, bijectivity, LP, and DP for generated S-boxes and comparison with benchmark approaches.

S-box	Balanced	Bijective	No. of fixed points	LP	DP
***S***_***1***_	Yes	Yes	0	0.0625	0.0156
***S***_***2***_	Yes	Yes	0	0.0625	0.0156
Zhang^8^	Yes	Yes	–	0.1320	0.0390
El-Latif^26^	Yes	–	–	0.1250	0.0313
Wang^34^	Yes	Yes	–	0.1250	0.0390
Alghafis^74^	Yes	No	2	0.0664	0.0156
F. Khan^42^	–	–	–	0.1406	0.0320
Ibrahim^68^	–	–	–	0.1172	0.0391
W. Gao^84^	Yes	Yes	0	0.125	0.0391
Siddiqui^70^	–	–	–	0.0625	0.0156
Silva-García^89^	No	No	1	–	–
Akimoski^88^	–	–	–	0.128	0.0390
AES	Yes	Yes	0	0.0625	0.0156
APA	Yes	Yes	0	0.0625	0.0156
Gray	Yes	Yes	0	0.0625	0.0156

All Boolean functions involved in the structure of the anticipated methodology to generate the confusion components satisfy the balance criteria, that is, $W_{f} \left( 0 \right) = 0$. The *XOR* operation among Boolean functions satisfies the bijection property. The proposed algorithm generates nonlinear components that meet the highest possible valuation for DP of *4/256*. Furthermore, the evaluated LP values of the intended and AES, APA, and Gray boxes are equivalent or superior to the state-of-the-art schemes shown in Table 7.

### Cryptanalytic analyses comparison

We executed numerous cryptanalytic analyses to measure the resistivity against diverse attacks. The reasoning for these findings are discussed in the supplementary data. Table 8 summarizes the findings of each investigation and their comparison with the available approaches.

**Table 8:** Cryptanalyses scrutinization of the generated S-boxes and comparison with benchmark approaches.

Analysis	*S*_*1*_	*S*_*2*_	Mazumdar^90^	AES	APA
Algebraic degree	7	7	7	7	7
Absolute indicator	32	32	–	32	32
Algebraic immunity	4	4	–	4	4
Composite algebraic immunity	4	4	–	4	4
Correlation immunity	0	0	–	0	0
Propagation criteria	0	0	–	0	0
Delta uniformity	4	4	–	4	4
Differential cryptanalysis	0.984	0.981	0.95	0.98	0.98
Differential power analysis	9.820	9.754	9.14	9.60	8.91
Transparency order	7.862	7.861	7.790	7.860	7.859
Coefficient variance	0.1097	0.1104	–	0.1113	0.1393

By investigating the outcomes in Table 8, the propagation criteria fulfill the assurity of diffusion properties in Boolean functions. The algebraic degree is sufficiently high to resist cryptanalytic attacks and is comparable with state-of-the-art results. Using the consequences of Tables 2, 3, we executed correlation attacks and found perfect correlation immunity. In our proposed model, the largest $\delta$ uniformity value is as low as in AES and APA structures, and the estimation of differential cryptanalytics for the assessed S-boxes is close to one. The estimated SNR and transparency order for the generated boxes were sufficiently high to provide resistivity against DPA attacks.

## Discussion

We demonstrated the integration of quantum states with the classical system for real-time environments by witnessing a point on which the quantum state reflects its symmetry, referred to as a quantum dot, rather than observing a superposition state on quantum machine into a definite state on classical system. We employ a conventional white-box, which has no impact on the quantumness features, to balance the functions. The produced sequence using proposed method for mono-bits and block frequency satisfies the intimacy of the ones fraction to almost one-half while ensuring the diffusion features in Boolean functions.

A multivalued cryptographic Boolean function employing a recurrent neural network was recently developed^91^. The network generates balanced confusion components with low linear and differential probability and a nonlinearity of 112. They train the net using rigorous limitations of activation function and the initialization of Mackey–Glass time series on the specified parameters, such as the time series' behavior becomes chaotic if τ grows from 17. In the experiment, 3000 learning samples over a specified period were analyzed to balance the parameters by mapping the intermission to itself. Each cycle yields a unique byte, resulting in 256 value vectors. If the generated S-box is not balanced, the system will repeat itself with new learning samples. Their method is ineffective for real-time applications due to space and execution constraints. Furthermore, they did not fulfill the avalanche and NIST criteria and did not undertake cryptanalytic investigations to validate the algorithm's efficacy against specific attacks, rendering it vulnerable to certain threats.

Similarly, with recent advances in quantum computations, the author proposed quantum spinning operators to develop confusion components^74^ with properties similar to traditional benchmarks, such as nonlinearity, BIC, SAC, and several others. Although the author employed quantum attributes to initiate the true random sequence, and the statistics are favorable, the approach is based on a random walk with Brownian motion. To overcome the challenge of superposition states into definite, as shown in Fig. 1d, e, the author launched a random walk with states on a classical system. The method focuses on random walk rather than true randomization characteristics to balance the confusion component of block cipher.

These methodologies produce better outcomes for confusion components, however, their integration with traditional systems in real-time environments is impractical because of the initial execution period and computational complexities. The developed model is simple and produces true random sequences, overcomes the aforementioned challenges, and produces superior outcomes than existing frameworks. It also has a higher resistance to hostile cryptographic attacks. The functions assessed in Tables 2, 3 are substantially compact without information loss and complex enough to be considered random. We observed from Tables 4, 5, 6, 7 that these functions maintain high resistivity in terms of linear cryptanalysis by:

- Maintaining the magnitude of the function's discrepancy lower and satisfying the 0/1 balance test,
- Satisfying the pairwise independence of the avalanche variables for a given set of avalanche vectors by complimenting a single plain bit, and
- Providing the least possible Hamming distance to the reference function from the set of all variable affine functions.

These functions validate the resistivity against differential and side-channel attacks while maintaining the diffusion characteristics. By witnessing the results in Table 8, the evaluated functions provide:

- Differential uniformity with small DP and $\delta$ value,
- Sufficiently high signal-to-noise ratio, and
- Immunity to correlation and algebraic attacks.

In comparison to recent neural network architectures and available quantum-assisted classical computation schemes for SPN network design, the proposed framework is easy to develop and deploy with favorable cryptographic characteristics, and has a high potential to resist statistical and differential attacks.

## Conclusion and future works

The security strength of block ciphers greatly relies on the confusion components to resist differential and linear attacks, and the threat of cryptanalysis using quantum classification by performing the reverse computation or executing brute force is one of the core issues of this decade. The produced design provides insights into quantum dots evolved from spin states to generate a truly random sequence for the confusion components, with high nonlinearity and low linear and differential probabilities, to overcome the quantum threats to block ciphers. To evaluate the efficiency of the proposed methodology, we compared the consequences of the intended S-boxes, based on widely accepted cryptographic and cryptanalytic measures, with benchmarks and state-of-the-art outcomes. The exhaustive contrast of these analyses showed that the algorithm is free of algebraic weakness with outstanding performance and provides robustness against linear and differential attacks.

We strongly believe that there is room for further improvements to envisioned structures with even better cryptographic properties. This model is designed for classical machines and can be used to modify the AES structure. The notions of the developed structure can be extended into a qubit model to protect the block ciphers against quantum computation threats. Reckonings of quantum dots in Bloch symmetry are possible when classical bits can be mapped into a qubit or in the form of quantum state(s).

## Supplementary Information

Supplementary Information 1.

## Data Availability

Correspondence and requests for materials should be addressed to H.M. Waseem or S.O. Hwang.

## References

[1] Zhang LY, Liu Y, Pareschi F, Zhang Y, Wong K-W, Rovatti R, Setti G (2017). On the security of a class of diffusion mechanisms for image encryption. IEEE Trans. Cybern..

[2] W. C. Barker and E. B. Barker, NIST Special Publication 800-67 Revision 1: Recommendation for the Triple Data Encryption Algorithm (TDEA) Block Cipher, (NIST, 2012).

[3] Advanced Encryption Standard (AES) (Federal Inf. Process, 2001).

[4] Daemen J, Rijmen V (2002). The Design of Rijndael: AES—The Advanced Encryption Standard, Heidelberg.

[5] Lai, X. & Massey, J. L. A proposal for a new block encryption standard. in *Proc. Workshop Theory Appl. Cryptograph. Techn.* 389–404 (1990).

[6] Fips Publication 46–3: Data Encryption Standard (DES) (NIST, 1999).

[7] Shannon CE (1949). Communication theory of secrecy systems. Bell Syst. Tech. J..

[8] Zhang T, Chen CLP, Chen L, Xu X, Hu B (2018). Design of highly nonlinear substitution boxes based on I-Ching operators. IEEE Trans. Cybern..

[9] Zhou Y, Panetta K, Agaian S, Chen CLP (2013). (n, k, p)-Gray code for image systems. IEEE Trans. Cybern..

[10] Khan M, Asghar Z (2018). A novel construction of substitution box for image encryption applications with Gingerbreadman chaotic map and S8 permutation. Neural Comput. Appl..

[11] He Y, Ying-Qian Z, Xin H, Xing-Yuan W (2021). A new image encryption algorithm based on the OF-LSTMS and chaotic sequences. Sci. Rep..

[12] Abd El-Latif AA, Ahmed A, Bassem AEA, Wojciech M, Carol F, Salvador EV (2020). Secure data encryption based on quantum walks for 5G Internet of Things scenario. IEEE Trans. Netw. Serv. Manag..

[13] Asgari-Chenaghlu M (2021). Cy: Chaotic yolo for user intended image encryption and sharing in social media. Inf. Sci..

[14] Abd El-Latif AA, Abd-El-Atty B, Venegas-Andraca SE (2019). A novel image steganography technique based on quantum substitution boxes. Opt. Laser Technol..

[15] Cho, J. Y. Linear cryptanalysis of reduced-round Present. In *Cryptographers’ Track at the RSA Conference*. (Springer, Berlin, Heidelberg, 2010).

[16] Heys HM (2002). A tutorial on linear and differential cryptanalysis. Cryptologia.

[17] Yu F, Xinhui G, Hanpeng L, Shihong W (2021). Differential cryptanalysis of image cipher using block-based scrambling and image filtering. Inf. Sci..

[18] Siddiqui N, Yousaf F, Murtaza F, Muhammad Ehatisham-ul-Haq M, Ashraf U, Alghamdi AM, Alfakeeh AS (2020). A highly nonlinear substitution-box (S-box) design using action of modular group on a projective line over a finite field. PLoS One.

[19] Xing C, Wang K (2021). Website information retrieval of web database based on symmetric encryption algorithm. J. Amb. Intell. Human. Comput..

[20] Zhang W, Pasalic E (2014). Highly nonlinear balanced S-Boxes with good differential properties. IEEE Trans. Inf. Theory.

[21] Piret G, Roche T, Carlet C (2012). PICARO—a block cipher allowing efficient higher-order side-channel resistance. Appl. Cryptogr. Netw. Secur..

[22] Bernardo-Gavito R, Ibrahim EB, Jonathan R, James S, Benjamin A, Hamzah S, Thomas MG (2017). Extracting random numbers from quantum tunnelling through a single diode. Sci. Rep..

[23] Ray B, Milenković A (2018). True random number generation using read noise of flash memory cells. IEEE Trans. Electron. Devices.

[24] Pironio S, Antonio A, Serge M, Boyer A, Dzmitry NM, Peter M, Steven O (2010). Random numbers certified by Bell’s theorem. Nature.

[25] Li D, Yu-Guang Y, Jing-Lin B, Jia-Bin Y, Juan X (2018). Controlled alternate quantum walks based quantum hash function. Sci. Rep..

[26] Abd AA, El-Latif BA-E-A, Amin M, Iliyasu AM (2020). Quantum-inspired cascaded discrete-time quantum walks with induced chaotic dynamics and cryptographic applications. Sci. Rep..

[27] Alghafis A, Waseem HM, Khan M, Jamal SS, Amin M, Batool SI (2020). A novel digital contents privacy scheme based on quantum harmonic oscillator and schrodinger paradox. Wirel. Netw..

[28] Arute F (2019). Quantum supremacy using a programmable superconducting processor. Nature.

[29] Alghafis A, Waseem HM, Khan M, Jamal SS (2020). A hybrid cryptosystem for digital contents confidentiality based on rotation of quantum spin states. Physica A.

[30] El-Latif A, Ahmed A, Bassem AEA, Salvador EVA, Wojciech M (2019). Efficient quantum-based security protocols for information sharing and data protection in 5G networks. Future Generat. Comput. Syst..

[31] Guo S, Zhao X, Zhang F, Wang T, Shi ZJ, Standaert F-X (2014). Exploiting the incomplete diffusion feature: a specialized analytical side-channel attack against the AES and its application to microcontroller implementations. IEEE Trans. Inf. Forensics Secur..

[32] Hu WH, Junnian W (2022). Cross subkey side channel analysis based on small samples. Sci. Rep..

[33] Nakahara Jr, J. , Barreto, P. S., Preneel, B., Vandewalle, J. & Kim, H. Y. SQUARE Attacks on Reduced-Round PES and IDEA Block Ciphers. In *IACR Cryptol. ePrint Arch., 68* (2001).

[34] Wang Y, Zhang Z, Zhang LY, Feng J, Gao J, Lei P (2020). A genetic algorithm for constructing bijective substitution boxes with high nonlinearity. Inf. Sci..

[35] Wang X, Nana G, Hongyu Z, Siwei W, Yingqian Z (2020). A new image encryption scheme based on coupling map lattices with mixed multi-chaos. Sci. Rep..

[36] Hussain I, Shah T, Mahmood H, Gondal MA (2013). A projective general linear group based algorithm for the construction of substitution box for block ciphers. Neural Comput. Appl..

[37] Zhou Y, Hua Z, Pun C, Philip Chen CL (2015). Cascade chaotic system with applications. IEEE Trans. Cybern..

[38] Behera PK, Gangopadhyay S (2021). Evolving bijective S-Boxes using hybrid adaptive genetic algorithm with optimal cryptographic properties. J. Amb. Intell. Human. Comput..

[39] Bolufé-Röhler A, Dania TV (2020). Machine learning based metaheuristic hybrids for S-box optimization. J. Ambient. Intell. Humaniz. Comput..

[40] Li Y-L (2015). Differential evolution with an evolution path: a DEEP evolutionary algorithm. IEEE Trans. Cybern..

[41] Shen M, Chen W-N, Zhang J, Chung HS-H, Kaynak O (2013). Optimal selection of parameters for nonuniform embedding of chaotic time series using ant colony optimization. IEEE Trans. Cybern..

[42] Khan MF, Saleem K, Alshara MA, Bashir S (2021). Multilevel information fusion for cryptographic substitution box construction based on inevitable random noise in medical imaging. Sci. Rep..

[43] Selçuk AA (2008). On probability of success in linear and differential cryptanalysis. J. Cryptol..

[44] Hermelin, M. & Nyberg, K. Linear cryptanalysis using multiple linear approximations. In *Advanced Linear Cryptanalysis of Block and Stream Ciphers* 29–53. (IOS Press, 2011).

[45] Chen J, Chen L, Zhou Y (2021). Universal chosen-ciphertext attack for a family of image encryption schemes. IEEE Trans. Multimedia.

[46] Li C, Preneel B, Paterson KG, Stebila D (2020). Improved interpolation attacks on cryptographic primitives of low algebraic degree. Selected Areas in Cryptography – SAC 2019: 26th International Conference, Waterloo, ON, Canada, August 12–16, 2019, Revised Selected Papers.

[47] Zhao K, Cui J, Xie Z (2017). Algebraic cryptanalysis scheme of AES-256 using Gröbner basis. J. Electr. Comput. Eng..

[48] Carlet C, Faugere JC, Goyet C, Renault G (2012). Analysis of the algebraic side channel attack. J. Cryptogr. Eng..

[49] Semenov A, Zaikin O, Otpuschennikov I, Kochemazov S, Ignatiev A (2018). On cryptographic attacks using backdoors for SAT. Proc. AAAI Conf. Artif. Intell..

[50] Sugita M, Mitsuru K, Hideki I (2006). Relation between the XL algorithm and Grobner basis algorithms. IEICE Trans. Fundam. Electron. Commun. Comput. Sci..

[51] Wentan YI, Linzhen LU, Chen S (2016). Integral and zero-correlation linear cryptanalysis of lightweight block cipher MIB. J. Electron. Inform. Technol..

[52] Zhang Y (2018). The unified image encryption algorithm based on chaos and cubic S-Box. Inf. Sci..

[53] Hua Z, Yicong Z (2016). Image encryption using 2D logistic-adjusted-sine map. Inf. Sci..

[54] Li C, Feng B, Li S, Kurths J, Chen G (2019). Dynamic analysis of digital chaotic maps via state-mapping networks. IEEE Trans. Circuits Syst. I Regul. Pap..

[55] Khan MF, Ahmed A, Saleem K (2019). A novel cryptographic substitution box design using Gaussian distribution. IEEE Access.

[56] Hua Z, Zhou Y (2016). Dynamic parameter-control chaotic system. IEEE Trans. Cybern..

[57] Preishuber M, Hütter T, Katzenbeisser S, Uhl A (2018). Depreciating motivation and empirical security analysis of chaos-based image and video encryption. IEEE Trans. Inf. Forensics Secur..

[58] Deng Y, Hanping H, Naixue X, Wei X, Lingfeng L (2015). A general hybrid model for chaos robust synchronization and degradation reduction. Inf. Sci..

[59] Wu X, Dawei W, Jürgen K, Haibin K (2016). A novel lossless color image encryption scheme using 2D DWT and 6D hyperchaotic system. Inf. Sci..

[60] Hua Z, Zhou B, Zhou Y (2019). Sine Chaotification model for enhancing chaos and its hardware implementation. IEEE Trans. Industr. Electron..

[61] Hua Z, Jin Fan, Binxuan Xu, Huang H (2018). 2D logistic-sine-coupling map for image encryption. Signal Process..

[62] Alawida M, Azman S, Je ST, Rami SA (2019). A new hybrid digital chaotic system with applications in image encryption. Signal Process..

[63] Cao C, Kehui S, Wenhao L (2018). A novel bit-level image encryption algorithm based on 2D-LICM hyperchaotic map. Signal Process..

[64] Tran, M. T., Bui, D. K. & Duong, A. D. Gray S-Box for Advanced Encryption Standard. In *2008 International Conference on Computational Intelligence and Security* 253–258, (2008).

[65] Tiwari N, Kumar A, Hemanth J, Fernando X, Lafata P, Baig Z (2019). Security effect on AES in terms of avalanche effect by using alternate S-box. International Conference on Intelligent Data Communication Technologies and Internet of Things (ICICI) 2018.

[66] Sahoo, O. B., Kole, D. K. & Rahaman, H. An optimized S-box for advanced encryption standard (AES) design. In *International Conference on Advances in Computing and Communications* 154–157 (IEEE, 2012).

[67] Dong Y, Geng Z, Yingjie M, Zhou P, Rui W (2022). A novel image encryption scheme based on pseudo-random coupled map lattices with hybrid elementary cellular automata. Inf. Sci..

[68] Ibrahim S, Abbas AM (2021). Efficient key-dependent dynamic S-boxes based on permutated elliptic curves. Inf. Sci..

[69] Mahmood Malik MS (2020). Generation of highly nonlinear and dynamic AES substitution-boxes (S-Boxes) using chaos-based rotational matrices. IEEE Access.

[70] Siddiqui N, Khalid H, Murtaza F, Ehatisham-Ul-Haq M, Azam MA (2020). A novel algebraic technique for design of computational substitution-boxes using action of matrices on Galois field. IEEE Access.

[71] Yang YG, Qing-Xiang P, Si-Jia S, Peng X (2015). Novel image encryption based on quantum walks. Sci. Rep..

[72] Yang YG, Qian-Qian Z (2016). Novel pseudo-random number generator based on quantum random walks. Sci. Rep..

[73] Waseem HM, Alghafis A, Khan M (2020). An efficient public key cryptosystem based on dihedral group and quantum spin states. IEEE Access.

[74] Alghafis A (2021). Quantum half and full spinning operator-based nonlinear confusion component. IEEE Access.

[75] Boixo S, Sergei VI, Vadim NS, Ryan B, Nan D, Zhang J, Michael JB, John MM, Hartmut N (2018). Characterizing quantum supremacy in near-term devices. Nat. Phys..

[76] Crutchfield JP (2012). Between order and chaos. Nat. Phys..

[77] Montiel O, Yoshio R, Cynthia O, Ajelet R (2019). Quantum-inspired acromyrmex evolutionary algorithm. Sci. Rep..

[78] Zeng M, Ee-Hou Y (2017). Discrete-time quantum walk with phase disorder: localization and entanglement entropy. Sci. Rep..

[79] Tsafack N, Jacques K, Bassem AEA, Abdullah MI, Kaoru H, Ahmed A, EL-Latif A (2020). Design and implementation of a simple dynamical 4-D chaotic circuit with applications in image encryption. Inform. Sci..

[80] Bernstein DJ, Tanja L (2017). Post-quantum cryptography. Nature.

[81] Khan M, Waseem HM (2018). A novel image encryption scheme based on quantum dynamical spinning and rotations. PLoS One.

[82] Cui L, Cao Y (2007). A new S-box structure named affine-power-affine. Int. J. Innov. Comput. Inform. Control.

[83] Kim J, Phan RC (2009). Advanced differential-style cryptanalysis of the NSA's skipjack block cipher. Cryptologia.

[84] Gao W, Idrees B, Zafar S, Rashid T (2020). Construction of nonlinear component of block cipher by action of modular group PSL(2, Z) on projective line PL(GF(2^8^)). IEEE Access.

[85] Qiao H, Kandel YP, Manikandan SK, Jordan AN, Fallahi S, Gardner GC, Manfra MJ, Nichol JM (2020). Conditional teleportation of quantum-dot spin states. Nat. Commun..

[86] Parvaz R, Zarebnia M (2018). A combination chaotic system and application in color image encryption. Opt. Laser Technol..

[87] Rukhin, A., Soto, J. & Nechvatal, J. A statistical test suite for random and pseudorandom number generators for cryptographic applications. *Proc. NIST* 1–164, (2010).

[88] Jakimoski G, Kocarev L (2001). Chaos and cryptography: block encryption ciphers based on chaotic maps. IEEE Trans. Circuits Syst. I Fundam. Theory Appl..

[89] Silva-García VM, Flores-Carapia R, Rentería-Márquez C, Luna-Benoso B, Aldape-Pérez M (2018). Substitution box generation using chaos: an image encryption application. Appl. Math. Comput..

[90] Mazumdar B, Mukhopadhyay D, Sengupta I (2013). Constrained search for a class of good bijective S-boxes with improved DPA resistivity. IEEE Trans. Inf. Forensics Secur..

[91] Abughazalah N, Asim L, Waseem HM, Majid K, Ammar SA, Iqtadar H (2022). Construction of multivalued cryptographic boolean function using recurrent neural network and its application in image encryption scheme. Artif. Intell. Rev..

