# Supplementary material for: Design of highly nonlinear confusion component based on entangled points of quantum spin states
Source: Sci Rep. 2023 Jan 19;13:1099. doi: 10.1038/s41598-023-28002-7 (PMC9852582; doi:10.1038/s41598-023-28002-7)
Supplement: Supplementary file 1 — Supplementary Information 1. [file 41598_2023_28002_MOESM1_ESM.docx]

Design of Highly Nonlinear Confusion Component Based on Entangled Points of Quantum Spin States

Muhammad Waseem Hafiz ^1, *^ and Seong Oun Hwang ^2, **^

^1^ Department of IT Convergence Engineering, Gachon University, South Korea
^2^ Department of Computer Engineering, Gachon University, South Korea
*[waseem@gachon.ac.kr](mailto:waseem@gachon.ac.kr), **[sohwang@gachon.ac.kr](mailto:sohwang@gachon.ac.kr)

**Abstract**

Cryptosystems are commonly deployed to secure data transmission over an insecure line of communication. To provide confusion in the data over insecure networks, substitution boxes are the solitary components for delivering a nonlinear mapping between inputs and outputs. A confusion component of a block cipher with high nonlinearity and low differential and linear approximation probabilities is considered secure against cryptanalysis. This study aims to design a highly nonlinear substitution-permutation network using the blotch symmetry of quantum spin states on the Galois field *GF* (2^8^). To observe the efficiency of the proposed methodology, some common and advanced measures were evaluated for performance, randomness, and cryptanalytics. The outcomes of these analyses validate that the generated nonlinear confusion components are effective for block ciphers and attain better cryptographic strength with a high signal-to-noise ratio in comparison to state-of-the-art techniques.

**Fundamental terminologies**

This section concisely explains the essential notions for developing the algorithm to generate the confusion components in conjunction with the main manuscript.

***Boolean Functions.*** A function $f\left(X_{n}\right)\in B_{n}$ with n tuples of elements from the vector space to refers to the Boolean function, where and $B_{n}$ are the set of Boolean functions in $n$ variables. $f\left(X_{n}\right)$ can be evaluated as:

,

where and $b=\left(b_{1}, b_{1},..., b_{n}\right)$. The algebraic degree of $f\left(X_{n}\right)$ ‘signified as $\deg\left(f\right)$’ is the utmost approximation of Hamming weight $\omega t\left(b\right)$, such that $\lambda_{b}\neq0$. If $\deg\left(f\right)=1$, $f$ is referred to as an affine function, and if it has a constant term equivalent to zero, then this affine function is referred to as a linear function.

***Galois Field.*** Digital data can be represented as a Galois vector, and the arithmetic properties of the Galois field are used for the scrambling and descrambling of data. For every prime $p$and positive integer $m$, each element in the Galois field $GF\left(p^{m}\right)$ can be distinctively signified by the linear combination of its basis $\left\{b_{0}, b_{1}, ..., b_{m-1}\right\}=\left\{\alpha^{0}, \alpha^{1}, ..., \alpha^{m-1}\right\}$ with coefficients from $GF\left(p\right)$, where $\alpha$ is the root of an irreducible polynomial $p\left(x\right)$ of degree $m$ over $GF\left(p\right)$, hence ^[1]^. For $p=2$ and $m=8$, there are a total of 16 primitive irreducible polynomials over $GF\left(2\right)$.

***Substitution box.*** The S-box is a collection of $m$ Boolean functions $F\left(X_{n}\right)=\left(f_{1}\left(X_{n}\right),...,f_{m}\left(X_{n}\right)\right)$ through the mapping of , where, $f_{1}, f_{2},..., f_{m}\in B_{n}$ are called constituent functions of *F*. The minimum algebraic degree of all nonzero linear combinations of constituent functions is denoted as , where $c=\left(c_{1}, c_{2},..., c_{m}\right)$ and .

### Evaluation criteria for s-boxes

The S-boxes correspond to a distinctive group of $n$ variable Boolean functions. Therefore, the subsequent properties are extensively acknowledged as essential criteria for evaluating the performance of the intended nonlinear components based on these Boolean functions.

***Balancedness and Bijectivity***. A Boolean function , is entitled as balanced if , that is, $HW\left(f\right)=2^{n-1}$ or $W_{f}\left(0\right)=0$. The consequence of the balance property is that if the magnitude of the function’s discrepancy is lower, then there will be a high possibility of achieving a low linear approximation probability. Hence, balancedness makes a Boolean function strong in terms of linear cryptanalysis.

The function , mapping each output to a unique input value, is referred to as a bijective. The bijection property can also be defined for each Boolean function $f_{i}$, as the XOR operation among $f_{j}$ Boolean functions that should satisfy 0*/*1 balance, where $\left(1\leq i\leq n\right)$ and $\left(2\leq j\leq n\right)$.

***Nonlinearity.*** The number of bits altered in the Boolean function’s truth table to reach the nearby affine function is referred to as nonlinearity. It signifies the least possible Hamming distance to the reference function $f\left(x\right)$ from the set of all variable affine functions $g\left(x\right)$ and is signified by $Nl\left(f\right)$ as:

$Nl\left(f\right)=\min\left\{x\in F_{2}^{n}|f(x)\neq g(x)\right\}$,

where *x* signifies the number of elements in the set, and normally, the Walsh spectrum is applied to calculate the nonlinearity of the set as $Nl\left(f\right)=2^{n-1}\left(1-2^{-n}\max_{w\in GF\left(2^{n}\right)} \left|WS_{f}\left(w\right)\right|\right)$,

where $GF\left(2^{n}\right)$ is the Galois field and the Walsh spectrum for the function can be calculated as:

$WS_{f}\left(w\right)=\sum_{x\in F_{2}^{n}} \left(-1\right)^{f\left(x\right)\oplus<x,w>}$,

where $<x,w>$ implies the dot product between vectors *x* and *w*. The correlation between the nonlinearity of the *n*-variable function and the Walsh transform for that function can also be evaluated directly as $Nl\left(f\right)=2^{n-1}-2^{\frac{n}{2}-1}$. For $GF\left(2^{8}\right)$, the upper bound value of nonlinearity is 120^[1]^.

***Strict Avalanche Criterion.*** SAC is the enactment of the avalanche effect and is satisfied when the output bits are flipped with a probability of 50 % by complimenting a single input bit. A function satisfies if $f\left(x\right)\oplus f\left(x\oplus\alpha\right)$ is balanced for $\alpha=1$. This norm assists a cipher to resist certain statistical attacks. When the S-box is used to generate the SPN architecture, the avalanche of deviations is affected by a single variation in the input of the network.

***Bit Independence Criterion.*** The independence between the avalanche variables is quantified with BIC. By complimenting a single plain bit, there will be pairwise independence of the avalanche variables for a given set of avalanche vectors. If the output bits of the two Boolean functions are $S_{j}$ and $S_{k}$ from the set $S_{1}, S_{2},..., S_{n}$ that fulfills the BIC, the SAC and nonlinearity must be $S_{j}\oplus S_{k}\left(j\neq k, 1\leq j, k\leq n\right)$. Hence, all functions must be nonlinear and satisfy the SAC if their corresponding S-box fulfills the BIC criterion.

***Differential Approximation Probability.*** DP refers to the variation in the output to a minute alteration in the input sequence. The input difference $\Delta x_{i}$ should uniquely map to an output difference $\Delta y_{i}$ to ensure uniformity in mapping probability for each *i*. To resist differential cryptanalysis, S-boxes must have differential uniformity. The maximum value of DP for an $n\times n$ S-box $F:2^{n}\to2^{n}$ should be as small as possible to resist such attacks. It can be evaluated as:

$DP_{F}=\max_{\Delta x \neq0, \Delta y} \frac{\left\{\Delta x\in X|f\left(x\right)\oplus f\left(x\oplus\Delta x\right)=\Delta y\right\}}{2^{n}}$,

where *X* is the set of all possible inputs, $\Delta x$ and $\Delta y$ are input and output differences, respectively, and $x\oplus\Delta x=\left(x_{1}\oplus\Delta x_{1}, x_{2}\oplus\Delta x_{2},..., x_{n}\oplus\Delta x_{n}\right).$

***Linear Approximation Probability.*** LP analyzed the imbalance between the input and output bits and determined the maximum disparity value of the event’s outcome^[2]^. LP has a maximum disparity if the input and output bits have equal parity. It can be defined as a cryptanalysis method that deals with the positions of several bits’ and has a distinction to observe a single bit position as in DP. The S-box is considered secure against linear cryptanalysis if it has a small linear probability^[3]^. For an $n\times n$ S-box $F:2^{n}\to2^{n}$, the LP can be defined as:

,

where and are the input and output parity bits in the corresponding masks, respectively.

***NIST statistical test suite.*** The validation of randomness in the bit stream generated with dot symmetry of entangled states over the 2-D plane is verified using the NIST statistical test suite (800-22)^[4]^. This entails multiple assessments to investigate the security of the anticipated design.

- The frequency (monobits) and block frequency tests are executed to observe the ratio of ones and zeros for the entire sequence and within M-bit blocks generated by the algorithm. The aim of these assessments is to determine whether the number of ones and zeros in the sequence and in the specific block(s) are approximately the same.
- The uninterrupted sequence of indistinguishable bits in the entire sequence and within *m*-bit blocks is determined using the run and longest run of ones investigations. These evaluations determine whether the fluctuation among substrings is fast or slow.
- The rank of the disjoint submatrices test is executed for the entire sequence to observe the linear dependence among fixed-length substrings of the sequence. The spectral test using the discrete Fast Fourier Transform in the test bench identifies the periodic features in the sequence that indicates the deviation from the notion of randomness. To observe the deviation from the predicted number of runs of ones at a given length, a periodic test is executed to reject those sequences, and an a-periodic assessment is performed to reject the sequences that exhibit too many fluctuations at the specified non-overlapping pattern.
- The universal statistical assessment is executed on the evaluated sequence to observe the matching pattern between the number of bits. This evaluation determines whether the sequence is considerably compact without information loss. To determine whether the generated sequence is complex enough to be considered random, a linear-complexity test is executed for the generated sequence to observe the length of its feedback register. A small feedback register infers the non-random estimations.
- The frequency of each overlapping pattern of *m*-bits in the entire sequence is analyzed with the serial test, and to relate the frequency of two adjacent overlapping blocks having length *m* and *m+1* in comparison to the expected outcomes for a random sequence, an approximate entropy test is executed.
- The cumulative sum trial is executed to observe the maximal excursion of the random walk. The purpose of this execution is to determine whether the sum of partial sequences occurring in the established sequence is large or small in comparison with the expected behavior of that sum for a random sequence. This walk should be near zero for random sequences.
- The trial of random excursion is evaluated to determine whether the number of visits to a state within the random walk exceeds the expected sequence. It entails the sequence of *n*-steps having a unit length taken randomly that begins and returns to the origin.
- To perceive the deviations from the estimated number of events of various states in a random walk, a random excursion variant event is evaluated to observe the number of times in which the specific state occurs in a cumulative sum random walk.

***Algebraic Degree.*** The maximum occurrence of a Boolean value in the truth table is entitled as an algebraic degree. It should be sufficiently high to resist cryptanalytic attacks, such as low-order approximation and differential attacks. It can be defined as:

$\deg\left(S_{r,c}\right)=\min\left(\deg\left(L_{a}\right)\right)$,

where $a=\cup_{i=1}^{n} 2^{i-1}$, $L_{a}$ is a set of all possible linear arrangements, and $n$ is the length of a Boolean function.

***Absolute Indicator.*** The absolute indicator is one of the measures used to determine the resistance offered by a Boolean function when used in the design of a cryptosystem. To evaluate the quality of the diffusion property for the block ciphers and the hash functions, the sum of the square indicator was additionally proposed. For all , we have , the absolute indicator of $f_{n}$ is defined as .

***Algebraic Immunity.*** Algebraic immunity reflects the resistance to algebraic attacks based on annihilators and is referred to as annihilator immunity. An annihilator of $f\in B_{n}$ is a function of $g\in B_{n}$, such that . These attacks are mounted in two phases.

- Find a system of equations $f_{i}\in B_{n}$ over that associates the secret bits with the public keystream, ciphertext, or plain text, etc.
- Solve the nonlinear system of equations to recover the secret bits. This method involves dropping the algebraic degree of the system. Numerous approaches can be used to achieve this^[5]^.

***Correlation Immunity.*** The resistance against correlation attacks among Boolean functions is measured by correlation immunity^[6]^. A function $f\in B_{n}$ has correlation immunity if its output is statistically independent of any input bits. An S-box is assumed to be $m^{th}$ order correlation immune if its Boolean function follows $1\leq HW\left(w\right)\leq m$ and $\hat{F}\left(w\right)=0$, where $\hat{F}\left(w\right)$ is the Walsh transform of input $B_{n}$.

***Propagation Criteria.*** The propagation criteria (*PC*) fulfill the assurity of diffusion properties in Boolean functions. The function $f$ with $n$-variable satisfies the *PC* pertaining to if the derivative function is balanced for every $b\in B_{n}$. The function satisfies $PC\left(k\right)$ if it fulfills the *PC* regarding the set , such that $HW\left(b\right)\leq k$.

***Differential (Delta) Uniformity.*** The largest outcome in the difference distribution table without counting $2^{n}$ values in the first row and column positions signifies the differential uniformity $\delta$. An S-box $\left(m,n\right)$ is $\delta$-uniform if $S_{m,n}\left(x\right)\oplus S_{m,n}\left(x\oplus v\right)=u$ provides $\delta$ solutions for every and . The low $\delta$ value validates strong robustness against differential cryptanalysis.

***Differential Cryptanalysis.*** Differential cryptanalysis exploits the largest outcome in the difference distribution table, including $2^{n}$ values in the first row and column positions. The estimation of differential cryptanalytics for the evaluated S-boxes should be high (close to 1) to provide resilience against these attacks.

***Differential Power analysis.*** Differential power analysis (DPA) refers to side-channel attacks. It exploits the variation in power characteristics consumed by circuitries to implement cryptographic algorithms. To measure the robustness against DPA attacks for S-boxes, we evaluated the variation in signal-to-noise ratio (SNR). A higher SNR is desirable to provide robustness against DPA attacks for the evaluated S-boxes.

***Transparency Order.*** The evaluation of the transparency order quantifies the resistance of the S-boxes against DPA attacks. For $f_{i}\in B_{n}$ over , the transparency order is defined as:

,

where $W_{D_{a} f}\left(u,v\right)$ is the Fourier transform of the sign function of the derivative of the Boolean function $f_{i}$ with respect to vector , that is, .

### Conclusion and future works

The security strength of block ciphers greatly relies on the confusion components to resist differential and linear attacks, and the threat of cryptanalysis using quantum classification by performing the reverse computation or executing brute force is one of the core issues of this decade. The produced design provides insights into quantum dots evolved from spin states to generate a truly random sequence for the confusion components, with high nonlinearity and low linear and differential probabilities, to overcome the quantum threats to block ciphers. To evaluate the efficiency of the proposed methodology, we compared the consequences of the intended S-boxes, based on widely accepted cryptographic and cryptanalytic measures, with benchmarks and state-of-the-art outcomes. The exhaustive contrast of these analyses showed that the algorithm is free of algebraic weakness with outstanding performance and provides robustness against linear and differential attacks.

We strongly believe that there is room for further improvements to envisioned structures with even better cryptographic properties. This model is designed for classical machines and can be used to modify the AES structure. The notions of the developed structure can be extended into a qubit model to protect the block ciphers against quantum computation threats. Reckonings of quantum dots in Bloch symmetry are possible when classical bits can be mapped into a qubit or in the form of quantum state(s).

**References**

1. W. Gao, B. Idrees, S. Zafar and T. Rashid, "Construction of Nonlinear Component of Block Cipher by Action of Modular Group PSL(2, Z) on Projective Line PL(GF(2^8^))," in *IEEE Access*, vol. 8, pp. 136736-136749, 2020.
2. R. Parvaz and M. Zarebnia,"A combination chaotic system and application in color image encryption," *Optics & Laser Technology*, 101, 30-41, 2018.
3. G. Jakimoski and L. Kocarev, "Chaos and cryptography: Block encryption ciphers based on chaotic maps", *IEEE Trans. Circuits Syst. I Fundam. Theory Appl.*, vol. 48, no. 2, pp. 163-169, Feb. 2001.
4. A. Rukhin, J. Soto and J. Nechvatal, "A statistical test suite for random and pseudorandom number generators for cryptographic applications", *Proc. NIST*, pp. 1-164, 2010.
5. B. Mazumdar, D. Mukhopadhyay and I. Sengupta, "Constrained search for a class of good bijective S-boxes with improved DPA resistivity", *IEEE Trans. Inf. Forensics Security*, vol. 8, no. 12, pp. 2154-2163, Dec. 2013.
6. X. Lai and J. L. Massey, "A proposal for a new block encryption standard", *Proc. Workshop Theory Appl. Cryptograph. Techn.*, pp. 389-404, 1990.

**Acknowledgment**

This work was supported by National Research Foundation of Korea (NRF) grants funded by the Korea government through Ministry of Science and ICT (MSIT) (2020R1A2B5B01002145).

**Author contributions**

H.M. Waseem conceived and conducted the experiments and S.O. Hwang analyzed the results. Both authors reviewed and approved the main and the supplementary manuscripts.

**Data availability**

Correspondence and requests for materials should be addressed to H.M. Waseem or S.O. Hwang.
